# Interleukin 17 in early invasive breast cancer

**DOI:** 10.3389/fonc.2023.1171254

**Published:** 2023-06-23

**Authors:** Marina Popović, Natalija Dedić Plavetić, Damir Vrbanec, Zlatko Marušić, Davor Mijatović, Ana Kulić

**Affiliations:** ^1^ Department of Oncology, University Hospital Center Zagreb, Zagreb, Croatia; ^2^ School of Medicine, University of Zagreb, Zagreb, Croatia; ^3^ School of Medicine, Juraj Dobrila University of Pula, Pula, Croatia; ^4^ Department of Pathology and Cytology, University Hospital Centre Zagreb, Zagreb, Croatia; ^5^ Department of Surgery, Division of Plastic, Reconstructive and Breast Surgery, University Hospital Centre Zagreb, Zagreb, Croatia; ^6^ Department of Oncology, Division of Experimental Oncology and Pathophysiology, University Hospital Centre Zagreb, Zagreb, Croatia

**Keywords:** interleukin-17, IL-17, breast cancer, cancer immunology, tumor-related inflammation, tumor microenvironment

## Abstract

**Introduction:**

Interleukin 17 (IL-17) has a key role in inflammatory responses. Increased serum concentrations of IL-17 have been reported in patients with different types of cancer. Some studies suggest antitumor activity of IL-17 while others speak in favor of its association with poorer prognosis. The lack of data on IL-17 behavior *in vivo* hinders the efforts to clarify the exact role of IL-17 in breast cancer patients and precludes the usage of IL-17 as potential therapeutic target.

**Methods:**

The study included 118 patients with early invasive breast cancer. The serum concentration of IL-17A was measured before surgery and during adjuvant treatment and compared with healthy controls. The correlation of serum IL-17A concentration and different clinical and pathological parameters, including IL-17A expression in the corresponding tumor tissue samples, was analyzed.

**Results:**

Significantly higher serum concentrations of IL-17A were found in women with early breast cancer before surgery, but also during adjuvant treatment in comparison to healthy controls. No significant correlation to tumor tissue IL-17A expression was observed. There was a significant postoperative decrease of serum IL-17A concentrations even in patients with relatively lower preoperative values. A significant negative correlation was found between serum IL-17A concentrations and the tumor estrogen receptor expression.

**Conclusion:**

The results suggest that the immune response in early breast cancer is mediated by IL-17A, particularly in triple-negative breast cancer. IL-17A-mediated inflammatory response subsides postoperatively, but IL-17A concentrations remain elevated compared to the values in healthy controls, even after the removal of the tumor.

## 1 Introduction

For many years breast cancer was considered as poorly immunogenic because, compared to tumors such are lung cancer, melanoma or kidney cancer, it has less TILs, lower mutational load and is less responsive to immunotherapy. A shift away from such an assumption was brought, among others, by the work done by Thorsson and colleagues, who defined six different tumor microenvironment (TME) immune subtypes using the genome and transcriptome data of 10,000 different tumors from the TGCA database, including breast cancer. Among the 944 breast cancer samples analyzed, none corresponded to the immunologically quiet group (1). Subsequently, Turan and colleagues noticed that transcriptional patterns of immune activity and immune suppression, i.e. activation of immunogenic cell death, IL23/Th17, “checkpoint” clusters, myeloid suppressor and regulatory T cells are simultaneously present in immune-active tumors and that their balance is in the background of the compensatory immune resistance (2). After the immune system is activated by tumor antigens and active inflammation is established in the TME, to avoid immune surveillance, instead of creating new ones, tumor cells pathologically strengthen the existing immune response regulation mechanisms that under physiological conditions control self-tolerance, homeostasis of myeloid cells, wound healing and cell death response (3). This leads to the activation of various oncogenic pathways that induce a change in the organization of the TME, so it becomes unfavorable for the anti-tumor immune response and eventually becomes qualitatively redirected to one that actually supports and promotes tumor growth (4). Inflammation is a necessary step in both tumor elimination and progression of malignant disease. The exact molecular mechanisms that underlie and determine whether the inflammatory process will lead to tumor elimination or promote tumor growth and dissemination are still not fully understood.

Interleukin 17A (IL-17A, referred to hereafter as IL-17 unless otherwise stated) was recognized as a central mediator of inflammation deeply involved in all phases of inflammatory events. Temporary and controlled IL-17 activity is part of the physiological immune response and tissue healing, while chronic IL-17 activity induces pathological responses that promote autoimmunity and oncogenesis (5). Antibodies targeting IL-17 have been successfully used to treat autoimmune inflammatory diseases such as multiple sclerosis, psoriasis, rheumatoid arthritis, and ankylosing spondylitis (6–8). Of the cells that secrete IL-17 in the TME, Th17 CD4+ lymphocytes and γδT cells predominate. Th17 CD4+ helper lymphocytes have the ability to transdifferentiate (9–11). Th17 cells, which express ROR γT and secrete IL-17 can simultaneously express Tbet and secrete IFN γ – Th17/Th1 hybrid cells or express Fox3 and secrete IL 10 – Th17/Treg hybrid cells (9, 11). Unlike Th1 and Th2, which probably represent definite and mutually exclusive cell populations, Th17 and Treg cells represent somewhat transitional forms that have the ability to change polarity under different conditions dictated by cytokine expression and tissue factors (11). Th17 cells obtained from the TIL population *in vitro* spontaneously transdifferentiated into Treg cells after TCR activation, which then lost their plasticity and could no longer be converted into Th17 cells, regardless of the presence of Th17 polarizing cytokines (12). It is not known whether similar changes occur *in vivo*. Unlike Th17 cells, γδT cells produce IL-17 by non-classical major histocompatibility and have properties of both innate immunity similar to NK cells and acquired immunity – variable γδTCR (13). Whether γδ T cells exhibit protumor characteristics similar to Treg cells or IFN γ mediated antitumor characteristics depends on the properties of the environment in which they are found. There is evidence that the production of IL-17 in γδ T cells takes place independently of TCR activation, while the secretion of IFN γ is enhanced after antigen exposure, so there are two phenotypes of γδ T cells: “innate” IL-17 γδ T and “acquired” IFN γ+ γδ T (14).

An elevated concentration of IL-17 was found in many tumors and was mostly associated with a worse prognosis (15). Zhu and colleagues were among the first to describe the expression of IL-17 in breast tumor tissue proposing a role of IL-17 in breast cancer invasion. Somewhat surprising, they found that macrophages were the main cells secreting IL-17, identified by cytology and CD68 staining (16). IL-17 directly induced breast cancer cell invasion independently of TNFα, which was inhibited by MMP selective antagonists. A few years later, analyzing the TME, Su and colleagues demonstrated that tumor cells form breast cancer cell lines and tumor-associated fibroblasts produce a pro-inflammatory cytokine milieu and secrete RANTES and MCP-1 that recruit Th17 cells in the TME (17). In 2013 Cochaud and colleagues analyzed the role of IL-17A and IL-17A producing TILs in human breast cancers. They found moderate to strong infiltration of IL-17-producing lymphocytes and macrophages in 20% of breast cancer samples, with a trend of association with estrogen receptor negative tumors (ER-), not reaching statistical significance, probably due to small numbers. Moreover, they showed that breast cancer cells harbor IL-17 receptors, and upon stimulation with IL-17 respond with the activation of ERK1/2 protein kinase pathway leading to cell proliferation and taxane resistance (18). Findings from several other studies supported the role of IL-17 in breast cancer progression and treatment resistance (19–24). Apart from having a direct effect on the breast cancer cell leading to the activation of ERK1/2 and JNK signaling pathways through phosphorylation of tumor progression locus 2 (TPL2) (25), it seems that IL-17 can exert its protumor effect in various different ways. IL-17 can promote tumor angiogenesis by inducing angiogenic factors CXCL8, MMP-2, MMP-9, and VEGF (26), it can direct the TME towards an immunosuppressive one by inhibiting apoptosis through NF-κB pathway activation (27) and by inducing the polarization of neutrophils towards a CD8+ T cell-suppressive phenotype (28). IL-17 can also induce the production of IL-6 and CCL20 in metastatic, but not in non-metastatic breast cancer tumor cells (23), thereby promoting inflammation and proliferation through IL-6 mediated STAT3 pathway activation (27). Even though metastatic and non-metastatic breast cancer tumor cells display different responses to IL17 *in vitro*, it does not mean IL-17 exerts no effect on non-metastatic cells *in vivo*. Analyzing gene-expression profile of non-metastatic breast cancer cell lines, that were either treated or not treated with IL17A, Benevides and colleagues identified 1,742 upregulated and 2,592 downregulated genes in the IL17-treated cells compared to untreated cells (23). Although the body of evidence speaking in favor of IL-17 contributing to poor prognosis in breast cancer patients is substantial, there is data showing anti-tumor activity of IL-17 (29, 30).

It seems that the effect of IL-17 in physiological conditions, as well as in the TME, can be two-fold, depending on the influence of various factors from the environment in which IL-17 is found (10). Knowing how the TME alters and evolves during disease progression, it is quite conceivable that the role of IL-17 is different in early and advanced breast cancer. The role of IL-17 in different types of tumors including breast cancer has so far been mostly investigated in *in vitro* cell models and human xenografts (31). Limited data on the concentration and behavior of IL-17 *in vivo* present a major obstacle in potential modulation of IL-17-mediated immune response in order to reduce its antitumor and enhance its protumor activity.

The aim of this study was to measure the serum IL-17A concentration in early invasive breast cancer patients before surgery and during adjuvant treatment and compare it to healthy controls, as well as to determine if there is a connection between the serum IL-17A concentration and the expression of IL-17A in the breast cancer tumor tissue.

## 2 Materials and methods

### 2.1 Patient evaluation

The research was conducted at the Clinical Hospital Center Zagreb and included 118 women who underwent surgery for early breast cancer from July 2015 to March 2018, and 63 healthy women who represented an age-matched control group. Only women with early invasive breast cancer, who were not candidates for neoadjuvant treatment, who had no prior history of cancer or an autoimmune disease, and who did not receive any immunosuppressive drugs at that time, were included in the research. Patients diagnosed with locally advanced or metastatic breast cancer and those for whom adequate follow-up was not possible, by taking a second blood sample, were excluded. Cohort characteristics are shown in **Table 1**. Pathology data, such as tumor type, histological and nuclear grade, surrogate intrinsic subtype, lymphovascular invasion and involvement of lymph nodes were obtained from the hospital information system. Estrogen and progesterone receptors, HER2 and Ki67 were determined by immunohistochemical staining using commercially available antibodies (Ventana, Tucson, AZ, USA). Estrogen and progesterone receptors were expressed as a percentage, and an expression equal to or greater than 1% was considered positive. HER2-positive breast cancer was defined by strong and complete immunohistochemical staining of the membrane of more than 10% of the cells (3+), and in case of inconclusive results (2+), *in situ* hybridization was performed in addition. Surrogate intrinsic subtypes were determined based on the expression of hormonal and HER 2 receptors and the proliferation marker Ki-67, according to the St. Gallen consensus from 2013 (32).

**Table 1:** Cohort characteristics (N=118).

		N	%
Type of surgery	Breast-sparing	73	61.90%
	Mastectomy	43	36.40%
	Other (biopsy)	2	1.70%
Menopause	No	39	33.10%
	Yes	79	66.90%
Castration	No	106	89.80%
	Yes	12	10.20%
Histological type	NST	93	78.80%
	Lobular	15	12.70%
	Mixed histology	6	5.10%
	Other	4	3.40%
Histological grade	1	9	8.20%
	2	67	60.90%
	3	34	30.90%
Nuclear grade	1	3	2.60%
	2	69	59.50%
	3	44	37.90%
Lymphovascular invasion	No	96	84.20%
	Yes	18	15.80%
HER-2 status	Negative (0,1+,2+)	101	85.60%
	Positive (3+ or 2+, ISH +)	17	14.40%
Surrogate subtype	Luminal A	45	38.10%
	Luminal B HER2 negative	46	39.00%
	Luminal B HER2 positive	9	7.60%
	HER2-enriched	8	6.80%
	Triple negative	10	8.50%
N group	N0	79	66.90%
	N1	29	24.60%
	N2	10	8.50%
Lymph node	Negative	79	66.90%
	Positive	39	33.10%
ER positive	No	18	15.3%
	Yes	100	84.7%
PR positive	No	30	25.4%
	Yes	88	74.6%
Chemotherapy	No	39	33.1%
	Yes	79	66.9%
Type of chemotherapy	Anthracycline	23	29.5%
	Anthracycline → taxane	45	57.7%
	Taxane	4	5.1%
	Other	6	7.7%
Anti-HER2 therapy	No	101	85.6%
	Yes	17	14.4%
Endocrine therapy	No	22	18.6%
	Yes	96	81.4%
Type of endocrine therapy	Tamoxifen	29	29.9%
	Aromatase inhibitors	64	66.0%
	Tamoxifen and aromatase inhibitors	4	4.1%
Radiotherapy	No	32	27.1%
	Yes	86	72.9%

All participants signed an informed consent form before entering the research. The research was conducted according to the Declaration of Helsinki and was approved by the local ethics committee.

### 2.2 Blood sample evaluation

Two blood samples were taken from each patient, one before surgery, and one after 3 cycles of chemotherapy or 3 months of endocrine therapy. Only one blood sample was taken from the healthy controls. The same inclusion criteria also applied to the control group. A 10 ml tube of whole blood was collected following standard procedures and centrifuged for 10 minutes at approximately 1000 G to separate the serum from the whole blood. Serum was then aliquoted into labeled cryovials and stored in an ultra-freezer at −80 °C. Serum IL-17A concentrations were determined using commercial sandwich ELISA kit (Human IL-17A Platinum ELISA, *BMS2017/BMS2017TEN*, eBioscience San Diego, CA, USA) according to the manufacturer’s instructions. Premixed human IL-17A standard was reconstituted by the addition of distilled water, generating a stock concentration of 200 pg/mL of IL-17A. Standard dilutions were prepared in 7 tubes (S1-S7), with the concentration of standard 1 = 100 pg/mL and serial dilutions 5 more times, generating seven points of standard curve. The limit of detection of human IL-17A was 0.5 pg/mL. Standard dilutions and samples were added to a 96-well, flat-bottomed, polystyrene microtiter plate, at the final volume of 100 μL in each strip. Each sample was assayed in duplicate. Biotin-Conjugate was added in each well. The microwell strips were then covered and incubated for 2 h at room temperature (RT). After incubation and washing, 100 μL of StreptavidinHRP was added to each well and left for another hour to incubate at RT. After incubation and washing, 100 μL of Substrate solution was added in wells and incubated for about 10 minutes at RT. Finally, 100 μL of stop solution was added to each well, and the results were read immediately after. The absorbance of each microwell was read on a spectro-photometer set to 450 nm. The results were expressed in pg/mL.

### 2.3 Tumor tissue evaluation

**Figure 1:**
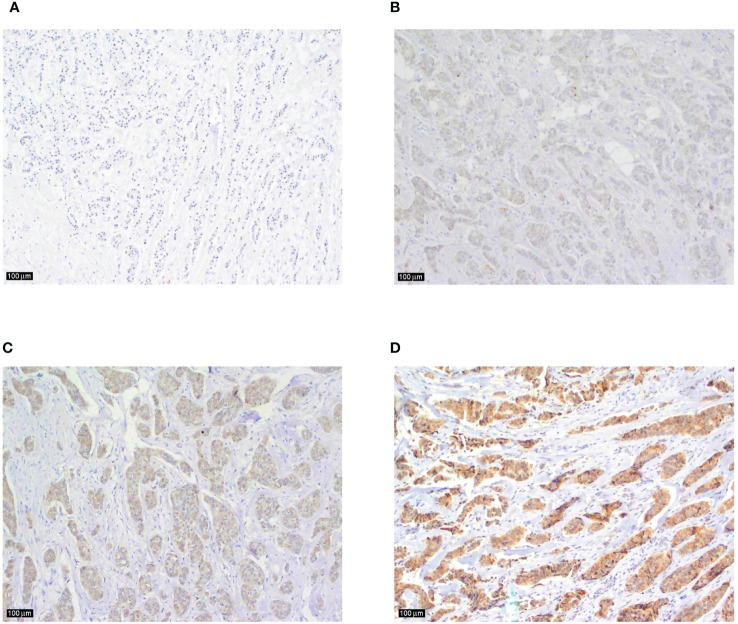
Examples of IL-17 tumor tissue expression in consecutive sections of breast cancer according to the staining intensity **(A)** tumor cells and surrounding tissue showing almost no staining (magnification x100), **(B)** an example of weak, and **(C)** moderate intratumoral staining intensity (magnification x100 in both sections), **(D)** strong immunohistochemical expression of IL-17 in breast cancer tumor tissue with no typical IL-17 producing immune cells visible in the peritumoral stroma (magnification x100).

Tumor tissue specimens in formalin-fixed, paraffin-embedded tissue blocks from 118 patients included in the research were retrieved from the archives of the Department of Pathology and Cytology, Clinical Hospital Centre Zagreb. Hemalaun eosin-stained, 4-µm-thick tissue sections were reviewed, and tissue sections from 97 patients with appropriate tumor cells and surrounding stroma were selected for immunohistochemical (IHC) analysis. Formalin-fixed (10%) paraffin-embedded tissue blocks (fixed at RT for 24-72 h) were cut into 4-µm sections. Antigen retrieval was performed using Dako PT link system with Dako Envision Flex Target Retrieval Solution, at low pH (6.0) for 20 min at 97°C, according to the manufacturer´s instructions (K800021, Dako, Agilent Tech., Santa Clara, CA, USA). The IHC was performed using Anti-IL-17A antibody (ab79056; Abcam, Cambridge, MA, USA) at 1:250 dilution for 45 min at RT and the Dako Envision FLEX system was used for visualization. Finally, the sections were counterstained using Mayer’s hematoxylin (S330930-2, Dako, Agilent Tech., Santa Clara, CA, USA) at RT for 5 min and mounted. Tonsil tissue was used as a positive control. Staining was examined by a specialist breast pathologist at University Hospital Centre Zagreb, Croatia. Staining results were evaluated semiquantitatively and scored according to the staining intensity: almost no staining (0), weak staining (1), moderate staining (2) and strong staining (3), as shown in **Figure 1**. Score 0 was considered as negative, while 1, 2 and 3 were considered as positive. The staining reaction was analyzed in invasive component and, if present, in *in situ* component, peritumoral stroma and surrounding healthy breast tissue.

### 2.4 Statistical analysis

The data were prepared using a Microsoft Office Excel spreadsheet calculator. Kolmogorov Smirnov test was used to analyze the distribution of continuous numerical values. Depending on the obtained data, corresponding non-parametric tests were applied. Categorical and nominal values were presented through corresponding frequencies and proportions. Continuous values were presented through medians and interquartile ranges. The differences between independent groups were analyzed using the Kruskal Wallis and Mann Whitney *U*-test. The differences in the serum IL-17A concentration before surgery and after three cycles of adjuvant chemotherapy or three months of endocrine therapy were analyzed using the Wilcoxon test. The correlation coefficients between IL-17A values and other clinical parameters were calculated. Spearman’s Rho test was used for the correlations of ordinal or scalar values. Kendall’s Tau-b test was used in case of ordinal-nominal correlations. p < 0.05 was considered statistically significant. All statistical tests were performed using IBM SPSS Statistics, version 25.0.

The results are shown in Tables and Figures. In the Figures, the results are shown using box plots with individual values included in the data analysis and marked as blank dots and squares. Outliers, i.e. values that were more than 1.5 times above the upper quartile, were not included in the data analysis and are marked as different shapes filled in black.

## 3 Results

### 3.1 Comparison of serum IL-17A concentration in early breast cancer before surgery and during adjuvant therapy and healthy controls

**Figure 2:**
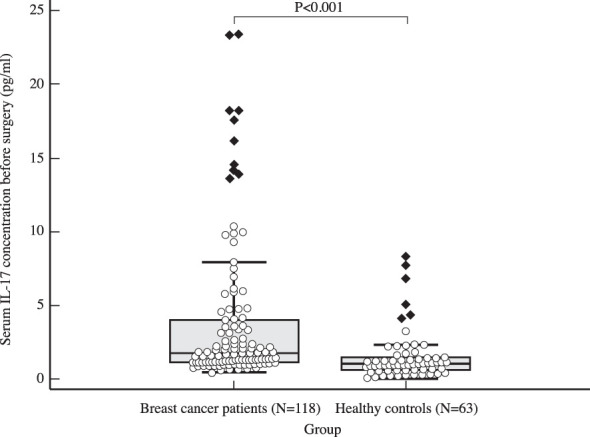
Comparison of serum IL 17 concentration in early breast cancer patients before surgery (N=118) and the control group (N=63) using Mann Whitney U test demonstrates a clear difference between the two groups, with significantly higher IL-17 levels observed in the study population (*P<*0.001).

**Figure 3:**
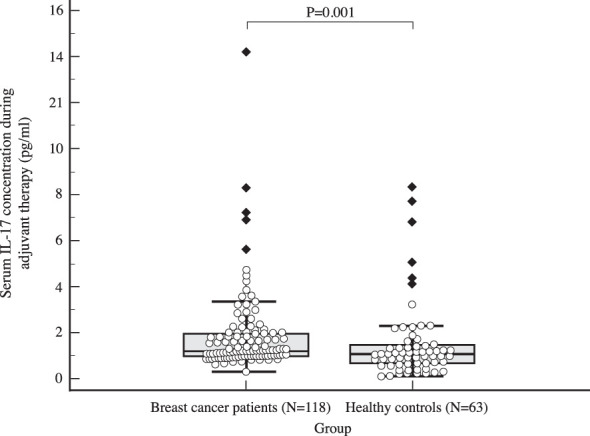
Comparison of serum IL 17 concentration in early breast cancer patients during adjuvant therapy (N=118) and the control group (N=63) using Mann Whitney U test, with a significantly higher postoperative IL-17 levels observed in the study population compared to the control group (*P=*0.001).

**Figure 4:**
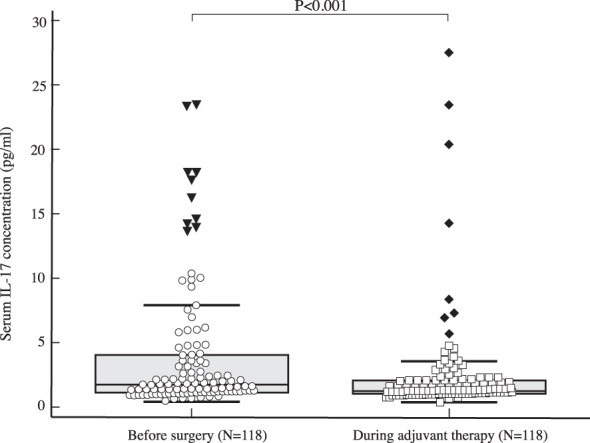
Comparison of serum IL 17 concentration in early breast cancer patients before surgery (N=118) and during adjuvant therapy (N=118) using Wilcoxon test shows a significant drop in IL-17 serum levels during adjuvant treatment compared to preoperative values (*P<*0.001).

The median serum IL-17A concentration in women with early invasive breast cancer before surgery and during adjuvant therapy was 1.82 pg/ml (1.15-4.72) and 1.25 pg/ml (1.00-2.05), respectively, while the median serum IL-17A concentration in healthy controls was 1.05 pg/ml (0.65-1.48). Compared to the control group, women with early invasive breast cancer had a significantly higher serum IL-17A concentration both before surgery (*P<*0.001) and during adjuvant therapy after surgery (*P=*0.001), as shown in **Table 2**, **Figures 2**, **3**. The difference between serum IL-17A concentrations in women with early invasive breast cancer preoperatively and after three cycles of adjuvant chemotherapy or three months of endocrine therapy is shown in **Table 3** and **Figure 4**. A significant decrease in the serum IL-17A concentration was observed after surgery during adjuvant therapy, compared to the preoperative values, (*P<*0.001).

Table 2Comparison of IL-17 serum concentration in women with early invasive breast cancer (cohort) before surgery and after surgery during adjuvant treatment and healthy women (control): Mann Whitney U test.

**Table:** 

		N	Min	Max	Centile		
					25.	Median	75.
Serum IL-17 concentration before surgery (pg/ml)	Cohort	118	0.43	224.20	1.15	1.82	4.72
	Control	63	0.08	8.32	0.65	1.05	1.48
Serum IL-17 concentration during adjuvant treatment (pg/ml)	Cohort	118	0.32	44.15	1.00	1.25	2.05
	Control	63	0.08	8.32	0.65	1.05	1.48

**Table:** 

	Mann-Whitney U	Z	P
Serum IL-17 concentration before surgery (pg/ml)	1902.00	-5.41	<0.001
Serum IL-17 concentration during adjuvant treatment (pg/ml)	2551.00	-3.47	0.001

Table 3Difference in the serum IL-17 concentration before surgery and after three cycles of adjuvant chemotherapy or three months of endocrine therapy: Wilcoxon test.

**Table:** 

	N	Min	Max	Centile		
				25.	Median	75.
Serum IL-17 concentration before surgery (pg/ml)	118	0.43	24.20	1.15	1.82	4.72
Serum IL-17 concentration during adjuvant treatment (pg/ml)	118	0.32	4.15	1.00	1.25	2.05

**Table:** 

	Z	P
Serum IL-17 concentration during adjuvant treatment (pg/ml)/ Serum IL-17 concentration before surgery (pg/ml)	-6.547	<0.001

### 3.2 Serum IL-17A concentration according to surrogate subtypes

**Figure 5:**
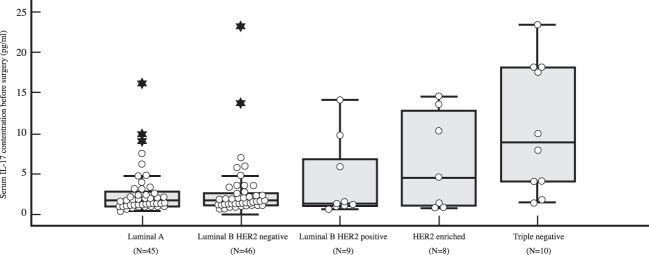
Differences in serum IL-17 concentration in early breast cancer patients before surgery depending on surrogate breast cancer subtype using Kruskal Wallis test demonstrate a significantly higher preoperative levels of IL-17 in TNBC (N=10) compared to HR+ breast cancer (*P=*0.001 for luminal A (N=45), *P=*0.003 for luminal B HER2 negative (N=46), and *P=*0.018 for luminal B HER2 positive (N=9), respectively).

**Figure 6:**
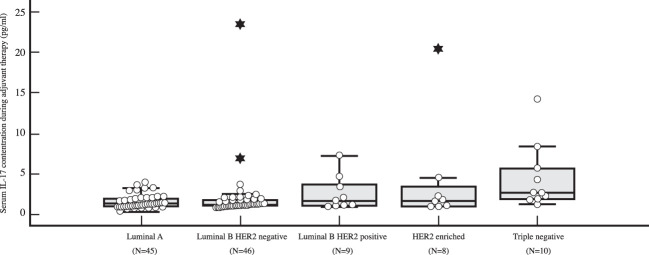
Differences in serum IL-17 concentration in early breast cancer patients during adjuvant therapy depending on surrogate breast cancer subtype using *post-hoc* Mann-Whitney U test, with a significantly higher serum IL-17 level (*P<*0.001) observed in TNBC (N=10) than in luminal A breast cancer (N=45).

The preoperative serum IL-17A concentration was significantly higher in triple negative breast cancer than in luminal breast cancer, i.e. luminal A (*P=*0.001), luminal B HER2 negative (*P=*0.003) and luminal B HER2 positive (*P=*0.018), as shown in **Table 4** and illustrated in **Figure 5**. A significantly higher serum IL-17A concentration was also found in triple negative subtype after surgery, during adjuvant treatment, in comparison to luminal A (*P<*0.001) and luminal B HER2 negative (*P=*0.001) subtypes, shown in **Table 4** and **Figure 6**. There was no significant difference in serum IL-17A concentration before surgery or during adjuvant therapy regarding HER 2 receptor positivity, **Table 5**.

Table 4Differences in serum IL-17 concentration before surgery and during adjuvant treatment depending on surrogate breast cancer subtype: Kruskal Wallis test and *post hoc* Mann Whitney U test.

**Table:** 

Surrogate breast cancer subtype		N	Min	Max	Centile		
					25.	Median	75.
Serum IL-17 concentration before surgery (pg/ml)	Luminal A	45	0.43	87.58	1.08	1.72	3.13
	Luminal B HER2 negative	46	0.60	224.20	1.16	1.74	3.55
	Luminal B HER2 positive	9	0.64	14.17	1.01	1.23	7.85
	HER2-enriched	8	0.78	34.20	1.00	7.45	14.31
	Triple negative	10	1.43	23.40	3.48	8.94	18.19
Serum IL-17 concentration during adjuvant treatment (pg/ml)	Luminal A	45	0.32	3.86	0.92	1.23	1.92
	Luminal B HER2 negative	46	0.83	44.15	1.01	1.22	1.87
	Luminal B HER2 positive	9	0.96	7.22	1.05	1.64	4.04
	HER2-enriched	8	0.97	20.40	0.99	1.67	3.94
	Triple negative	10	1.23	14.20	1.81	2.60	6.30

**Table:** 

	Kruskal-Wallis H	df	P
Serum IL-17 concentration before surgery (pg/ml)	12.631	4	0.013
Serum IL-17 concentration during adjuvant treatment (pg/ml)	14.737	4	0.005

**Table:** 

Surrogate breast cancer subtype	*Post-hoc* analysis: Mann-Whitney U test	
	Serum IL-17 concentration before surgery (pg/ml)	Serum IL-17 concentration during adjuvant treatment (pg/ml)
Luminal A *vs*. Luminal B HER2 negative	0.446	0.378
Luminal A *vs*. Luminal B HER2 positive	0.807	0.131
Luminal A *vs*. HER2-enriched	0.149	0.176
Luminal A *vs*. Triple negative	0.001	<0.001
Luminal B HER2 negative *vs*. Luminal B HER2 positive	0.474	0.363
Luminal B HER2 negative *vs*. HER2-enriched	0.306	0.450
Luminal B HER2 negative *vs*. Triple negative	0.003	0.001
Luminal B HER2 negative *vs*. HER2-enriched	0.290	0.923
Luminal B HER2 negative *vs*. Triple negative	0.018	0.102
HER2-enriched *vs*. Triple negative	0.477	0.155

Table 5Differences in serum IL-17 concentrations before surgery and during adjuvant treatment depending on HER2 status: Mann Whitney U test.

**Table:** 

HER-2 status		N	Min	Max	Centile		
					25.	Median	75.
Serum IL-17 concentration before surgery (pg/ml)	Negative (0.1+.2+)	101	0.43	224.20	1.16	1.82	4.02
	Positive (3+ or 2+ ISH+)	17	0.64	34.20	1.01	1.53	11.97
Serum IL-17 concentration during adjuvant treatment (pg/ml)	Negative (0.1+.2+)	101	0.32	44.15	0.99	1.23	2.01
	Positive (3+ or 2+ ISH+)	17	0.96	20.40	1.01	1.64	3.93

**Table:** 

	Mann-Whitney U	Z	P
Serum IL-17 concentration before surgery (pg/ml)	837.500	-0.161	0.872
Serum IL-17 concentration during adjuvant treatment (pg/ml)	704.000	-1.184	0.236

### 3.3 Tissue expression of IL-17A

The expression of IL-17A was significantly higher in the tumor tissue and the peritumoral stroma than in the surrounding normal breast tissue, **Table 6**. There was no statistically significant correlation of IL-17A serum concentration and expression of IL-17A in the tumor tissue, as shown in **Table 7**.

**Table 6:** Comparison of intratumoral expression of IL-17 (invasive component. *in situ* component. peritumoral stroma) with normal tissue: Spearman’s correlation coefficient.

		IL-17 IHC normal tissue
IL-17 IHC invasive and *in situ* component	Correlation coefficient	0.441
	*P*	<0.001
	N	71
IL-17 IHC peritumoral stroma	Correlation coefficient	0.426
	*P*	<0.001
	N	72

**Table 7:** Correlation of serum IL-17 concentration and tumor tissue expression: Spearman correlation coefficients Rho.

		Serum IL-17 concentration before surgery (pg/ml)	Serum IL-17 concentration during adjuvant treatment (pg/ml)
IL-17 IHC invasive and *in situ* component	Rho	-0.024	0.036
	*P*	0.816	0.726
	N	95	95
IL-17 IHC peritumoral stroma	Rho	0.054	0.111
	*P*	0.603	0.282
	N	96	96
IL-17 IHC normal tissue	Rho	-0.030	-0.072
	*P*	0.799	0.543
	N	73	73

### 3.4 Correlation of serum IL-17A concentration and tissue expression with other clinical and pathohistological parameters

A statistically significant negative correlation of serum IL-17A concentration and the expression of estrogen receptors and the use of hormone therapy was observed before, but also after surgery during adjuvant treatment, while a positive correlation was observed with the use of chemotherapy. The concentration of IL-17A in serum during adjuvant therapy was also significantly positively correlated with the histological grade, nuclear grade, tumor size and the involvement of axillary lymph nodes. Intratumoral expression of IL-17A significantly positively correlated only with the use of chemotherapy. Meanwhile, a significantly positive correlation was found between the peristromal IL-17A expression and tumor nuclear grade, proliferation index Ki-67 and the use of chemotherapy, while a negative correlation was observed with age and menopause. The expression of IL-17A in normal tissue significantly negatively correlated only with age. Data are shown in **Table 8**.

Table 8Correlation coefficients between serum IL-17 concentration and other clinical and pathohistological parameters: Spearman’s correlation coefficients Rho or Kendall’s correlation coefficients Tau_b (in cases of ordinal-nominal comparisons).

**Table:** 

		Serum IL-17 concentration before surgery (pg/ml)	Serum IL-17 concentration during adjuvant treatment (pg/ml)	IL-17 IHC invasive and *in situ* component	IL-17 IHC peritumoral stroma	IL-17 IHC normal tissue
Age et diagnosis (years)	Rho	-0.179	-0.113	-0.109	-0.342	-0.275
	*P*	0.052	0.224	0.291	0.001	0.019
	N	118	118	96	96	73
Menopause	Tau_b	-0.139	-0.066	-0.123	-0.206	-0.134
	*P*	0.067	0.383	0.234	0.044	0.254
	N	118	118	96	96	73
Castration	Tau_b	-0.024	-0.026	0.73	0.044	0.038
	*P*	0.755	0.735	0.479	0.665	0.744
	N	118	118	96	96	73
Histological grade	Rho	0.112	0.232	0.72	0.123	0.006
	*P*	0.243	0.015	0.504	0.249	0.959
	N	110	110	89	90	67
Nuclear grade	Rho	0.029	0.189	0.141	0.256	0.195
	*P*	0.760	0.042	0.175	0.013	0.101
	N	116	116	94	94	72
Lymphovascular invasion	Tau_b	0.144	0.103	0.183	0.036	0.116
	*P*	0.062	0.184	0.079	0.732	0.324
	N	114	114	93	93	72
Tumor size (cm)	Rho	0.167	0.224	0.018	0.149	0.101
	*P*	0.071	0.015	0.838	0.148	0.397
	N	118	118	96	96	73
N group	Rho	0.095	0.174	0.169	0.168	-0.072
	*P*	0.306	0.060	0.100	0.102	0.543
	N	118	118	96	96	73
Positive lymph nodes	Tau_b	0.100	0.151	-0.119	0.167	-0.056
	*P*	0.188	0.047	0.484	0.102	0.634
	N	118	118	37	96	73

**Table:** 

		Serum IL-17 concentration before surgery (pg/ml)	Serum IL-17 concentration during adjuvant treatment (pg/ml)	IL-17 IHC invasive and *in situ* component	IL-17 IHC peritumoral stroma	IL-17 IHC normal tissue
ER (%)	Rho	-0.215	-0.275	0.185	-0.073	0.135
	P	0.019	0.003	0.071	0.477	0.256
	N	118	118	96	96	73
PR (%)	Rho	-0.034	-0.114	0.087	-0.150	0.007
	P	0.711	0.220	0.399	0.144	0.956
	N	118	118	96	73	73
HER-2 status	Rho	0.015	0.109	0.082	0.101	0.000
	P	0.873	0.238	0.427	0.325	1.000
	N	118	118	96	96	73
Ki-67 (%)	Rho	0.120	0.147	0.084	0.249	0.148
	P	0.201	0.115	0.423	0.015	0.213
	N	116	116	94	95	73
Chemotherapy	Tau_b	0.151	0.176	0.205	0.319	0.062
	P	0.046	0.021	0.045	0.002	0.594
	N	118	118	96	96	73
Anti-HER therapy	Tau_b	0.012	0.090	0.082	0.101	0.000
	P	0.872	0.236	0.427	0.323	1.000
	N	118	118	96	96	73
Endocrine therapy	Tau_b	-0.244	-0.230	0.202	0.048-0.063	0.055
	P	0.001	0.003	0.058	0.534	0.640
	N	118	118	96	96	73
Radiotherapy	Tau_b	0.011	0.133	-0.033	-0.002	-0.157
	P	0.884	0.081	0.751	0.986	0.181
	N	118	118	96	96	73

## 4 Discussion

Breast tumors represent a heterogeneous group of diseases with different histological and molecular characteristics, clinical presentation, response to treatment and prognosis. The complex and dynamic interplay between immune system and breast cancer cells still hasn’t been fully elucidated. Not many studies have analyzed the serum IL-17 concentration in breast cancer, but those that did, repeatedly indicate that there is a significant increase compared to the healthy women, even in early breast cancer (27, 33–35). In accordance with previous research, data analysis from this research revealed a significantly higher serum concentration of IL-17A in the serum of women with early breast cancer at diagnosis, but also after surgical removal of the tumor during adjuvant treatment compared to the control group. A statistically significant decrease in the level of IL-17A in the serum of women with early breast cancer was recorded during adjuvant treatment after surgery compared to the values before surgery. The results support the fact that the immune response in early breast cancer is mediated, among other, by cells that secrete IL-17A, and that after the removal of the tumor, even though the reaction mediated by IL-17A subsides, serum IL-17A level remains elevated compared to the healthy population.

Significantly higher preoperative serum IL-17A levels were recorded in women with triple-negative breast cancer compared to hormone-dependent cancer. The results are similar to that found by Liu et al., observing the serum levels of different cytokines, including IL-17, in early breast cancer patients (35). Earlier studies indicate that the level of IL-17 is significantly increased in ER negative or triple negative breast cancer tissue compared to other surrogate subtypes. An analysis of data from the TCGA database showed that high expression of ER decreases the level of IL-17A, IL-17C and IL-17F, and increases the level of IL-17E, which suppresses signaling mediated by IL-17. ER-related gene expression was negatively associated with the expression of genes involved in IL-17 signaling. Moreover, increased expression of PD-L1 was associated with ER negative status and increased expression of genes involved in IL-17 signaling (36). By measuring the serum IL-17 level in ER negative breast cancer with low and high PD-L1, Yun Feng Ma et al. found that the serum IL-17 level was significantly higher in ER negative breast cancer with high PD-L1 expression (24). In our research, the serum IL-17A level significantly negatively correlated with the tumor ER expression before surgery, but also during adjuvant treatment. As expected, the serum IL-17A concentration negatively correlated with hormonal and positively with chemotherapy treatment. Since chemotherapy is the standard treatment in triple-negative early breast cancer, when considering the plausible cause of elevated IL-17A level during adjuvant treatment, it should be kept in mind that chemotherapy causes tissue injury that is commonly accompanied by inflammation (37). A trend of lower serum IL-17A levels was recorded in elderly patients and in menopausal patients before surgery. This is possible due to a higher incidence of hormone-dependent breast cancer in this group of patients (38), but could also be the result of the effects of aging on the immune system. Significantly higher serum IL-17A concentration in triple negative subtype was also recorded during adjuvant treatment after surgery compared to luminal A breast cancer. The recently published data confirm the association of IL-17 with the *MEGF11* gene (multiple epidermal growth factor like domains 11), whose expression is significantly higher in the tumor tissue of triple-negative breast cancer patients who experienced relapse (39). A study followed in which Tsai Y-F et al. attempted to elucidate the role of IL-17 in triple-negative breast cancer relapse. It turned out that in triple-negative breast cancer, IL-17 promotes the migratory activity of tumor cells and directs the immune landscape, creating conditions in the microenvironment that support dissemination (40). A relatively small number of triple-negative breast cancer patients were included in this research. More patients and a longer follow-up will be needed to verify the possible influence of serum IL-17A levels preoperatively or during treatment on triple negative breast cancer disease recurrence.

The results showed no correlation between the expression of HER2 receptor and serum IL-17A concentration. Accordingly, serum IL-17A level in HER2 positive breast cancer did not differ significantly from that in other surrogate breast cancer subtypes. A somewhat contrasting result was obtained by Liu and colleagues, who found that, in addition to TNBC, the level of IL-17 was also elevated in HER2 positive breast cancer patients, when compared to luminal tumors. It should be noted though, that their study was enriched with HER2 positive patients (41.7%) (35). Similar results were found in a different study evaluating Th17 cytokine profile in breast cancer patients, showing elevated IL-17 levels in aggressive molecular subtypes, including HER2 positive breast cancer (41). Although HER2+ tumors are more common among patients with inflammatory breast cancer (36) and, as triple negative, HER2+ breast cancer is typically associated with more pronounced local inflammation, no connection was found between HER-2 positivity and tissue expression or serum level of IL-17, even in earlier studies (24, 42). Although on a small number of HER2+ metastatic breast cancer patients, Horlock et al. detected that HER2+ breast cancer patients had a significantly lower level of circulating Th17 cells at the expense of Treg cells, compared to the healthy population. Interestingly, in metastatic disease, trastuzumab therapy changed the Treg : Th17 ratio in favor of Th17 cells, while no such effect was observed in early HER2+ breast cancer during adjuvant treatment (43). The level of IL-17 and CD4+Th17 lymphocytes is not always mutually equivalent. As Coffelt et al. demonstrated, the depletion of neutrophils in a mouse model reduced the number of metastases in the early breast cancer. In the studied model, the expansion and phenotype of neutrophils directly depended on the IL-17→G-CSF signaling cascade. After depletion of both CD4+ and γδ T lymphocytes, the authors concluded that it was precisely the depletion of γδ T lymphocytes that led to a significant decrease of IL-17 and G-CSF concentration and consequently to the reduction of the circulating neutrophils (28). The fact that the biological activity of IL-17 is not necessarily equal to the IL-17 mRNA level or the number of cells secreting IL-17 should also be taken into account. Since the biological activity of IL-17 can be stimulated or inhibited by different mediators. For example, it can be enhanced by TNF-α, IL-1, GM-CSF and IFN-γ, but also diminished by IL-25 (IL-17E), IL-17 autoantibodies and soluble IL-17R (6, 44).

No significant correlation was found between the serum IL-17A concentration before surgery and characteristics such as the tumor histological type, histological and nuclear grade, positive lymphovascular invasion, proliferation index, tumor size and involvement of lymph nodes. A similar result was obtained by Chen et al. looking at the tumor tissue expression of IL-17 (42). Research on tumor infiltrating lymphocytes (TILs) in early breast cancer as a surrogate marker of acquired immunity suggests that the key role in the formation of the immune response in early breast cancer is played by the biological characteristics of the tumor, and not the volume of the disease. Analyzing 897 triple-negative breast cancer samples, Pruneri et al. found no significant associations between TIL levels and clinicopathological characteristics such as the tumor size, lymph node involvement, or lymphovascular invasion (45). Knowing that early breast cancer is a more homogeneous disease than disseminated breast cancer, the modulation of the immune response could be more effective in the long term. Observing the serum IL-17A concentration after surgery during adjuvant treatment, a significant positive correlation was found with histological grade, nuclear grade, tumor size and lymph node involvement. Intuitively, this result does not surprise considering that the mentioned tumor characteristics are important prognostic variables used in the clinical assessment of the disease recurrence risk and specific survival in operable breast cancer (46).

The intratumoral expression of IL-17A was significantly higher than the expression of IL-17A in healthy breast tissue. The main site of IL17A expression was breast cancer tumor tissue and to a lesser degree peritumoral stroma. This is somewhat different from the staining results observed in earlier studies looking at tumor tissue IL-17 expression, in which the main site of IL-17 secretion were, as expected, inflammatory cells in the tumor environment, primarily lymphocytes and macrophages (18, 26, 42). However, the autocrine origin of inflammatory cytokines is well documented in different tumors (47, 48). In the research conducted by Mombelli et al., studying the signaling pathways that activate IL-17A and IL-17 E in breast cancer cells using the method of reverse transcription coupled with quantitative polymerase chain reaction (RT qPCR), the expression of IL-17RA/RC and IL-17RA receptors was significantly higher in tumor than in normal breast tissue, backing the presence of active IL-17A and IL-17E signaling in breast cancer cells (22). Although they were not able to detect IL-17E transcripts in the tumor cell lines, previous reports showed IL17B to be produced by malignant cells, inducing tumorigenesis in an autocrine manner (49, 50). In the work recently done by Ali et al. (51), studying the IHC expression of IL-17 in 137 benign and malignant breast lesions, the main site of IL17 expression were malignant cells themselves. Zhang et al. (52) and Al-Samadi et al. (53) observed the same intratumoral IL-17 staining patterns in breast cancer tissue and colorectal cancer tissue samples, respectively. Either way, consideration will have to be given to the robustness of the IL-17 IHC testing in the future. Characterization of the sensitivity and specificity of different IL17A antibodies will have to be done to reduce the potential cross-reactivity with other IL-17 isoforms, since different IL-17 isoforms can activate different signaling pathways even within the same cancer type.

Unexpectedly, we found no statistically significant correlations between the serum IL-17A level before surgery or during adjuvant treatment and IL-17A tissue expression. This could perhaps be due to methodology limitations. Another plausible explanation could be in line with the research conducted by Azizi et al. who, using mRNA techniques, profiled the phenotype of 45,000 immune cells from 8 breast cancer and corresponding healthy breast tissue, lymph node and peripheral blood samples, creating a kind of “immunological atlas” of the breast cancer microenvironment. They observed a significant distinction in the immune cell phenotype of different tissue compartments. Similar immune cell clusters were found in tumor tissue and surrounding normal breast tissue that differed from those found in lymph nodes and peripheral blood (54). This could mean that immune cell biomarkers in peripheral blood do not necessarily reflect the composition of immune cells in the TME.

Antibodies that target IL-17 are successfully used in the treatment of autoimmune diseases. Secukinumab was the first IL-17 antibody approved by the US Food and Drug Administration (FDA) in 2015 for the treatment of psoriasis (55). Following excellent efficacy secukinumab has shown in clinical trials, two other humanized monoclonal IL-17A antibodies ixekizumab and netakimab, and an IL-17A receptor antagonist brodalumab, gained FDA approval for treatment of psoriasis and axial spondiloarthritis (56–58). There are several preclinical studies showing efficacy of IL-17A antibodies in the treatment of cancer. In colon cancer and melanoma higher IL-17 expression was associated with the resistance to anti-PD-1 therapy (59, 60). In a preclinical model of human gastric cancer, Nagaoka et al. successfully eradicated the tumor using combined PD-1 and IL-17A blockade (61). The role of IL-17A blockade in breast cancer treatment is yet to be examined. Used alone or more likely as a combination therapy, IL-17A antibodies could potentially help to overcome resistance to checkpoint inhibitors or chemotherapy in metastatic, but even more in adjuvant setting. Given the specific and sometimes contradictory role of IL-17 in TME it is likely that IL-17–based therapy will need to be tailored if not to a single patient at least to a particular cancer type and the extent of the cancer spread.

## 5 Conclusion

In conclusion, the serum IL-17A concentration in early breast cancer patients is significantly higher than in the healthy individuals and dominantly depends on the surrogate breast cancer subtype and not on the histological subtype or local extension of the disease. After tumor removal, during adjuvant treatment, the serum IL-17A level significantly drops, but nevertheless remains elevated compared to the healthy controls. IL-17A expression in tumor tissue and peritumoral stroma is significantly higher than in healthy breast tissue but does not correlate with IL-17A serum values either before surgery or during adjuvant treatment.

## Data availability statement

The original contributions presented in the study are included in the article/supplementary material. Further inquiries can be directed to the corresponding author.

## Ethics statement

The studies involving human participants were reviewed and approved by University Hospital Centre Zagreb Ethics Committee. The patients/participants provided their written informed consent to participate in this study.

## Author contributions

MP, NP, DV, and AK made substantial contributions to the conception and design of the work. MP, DM, ZM, and AK were responsible for the acquisition of data for the work. MP and NDP organized the database. AK and ZM analyzed the data while MP, NP, and DV interpreted the data. MP drafted the work, and ZM and AK wrote parts of the manuscript. NP revised the work critically. All authors provided approval for publication. All authors agree to be accountable for all aspects of the work in ensuring that questions related to the accuracy or integrity of any part of the work are appropriately investigated and resolved. All authors contributed to the article.
